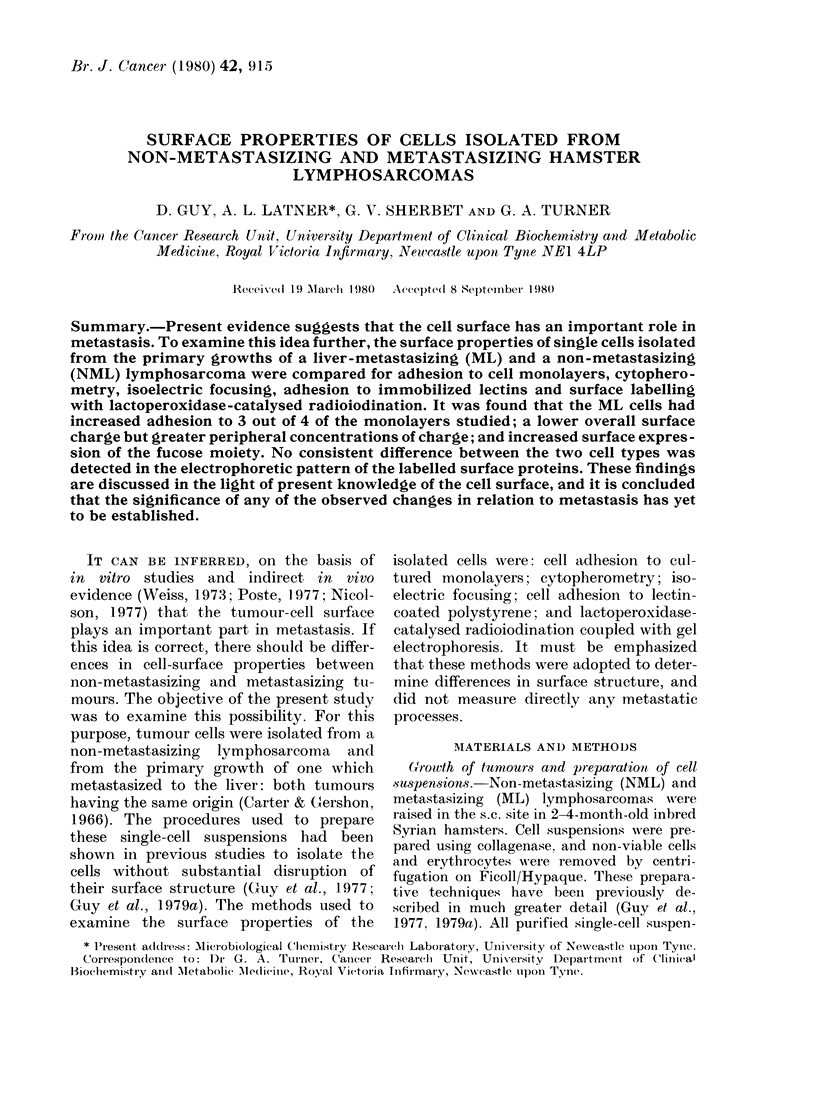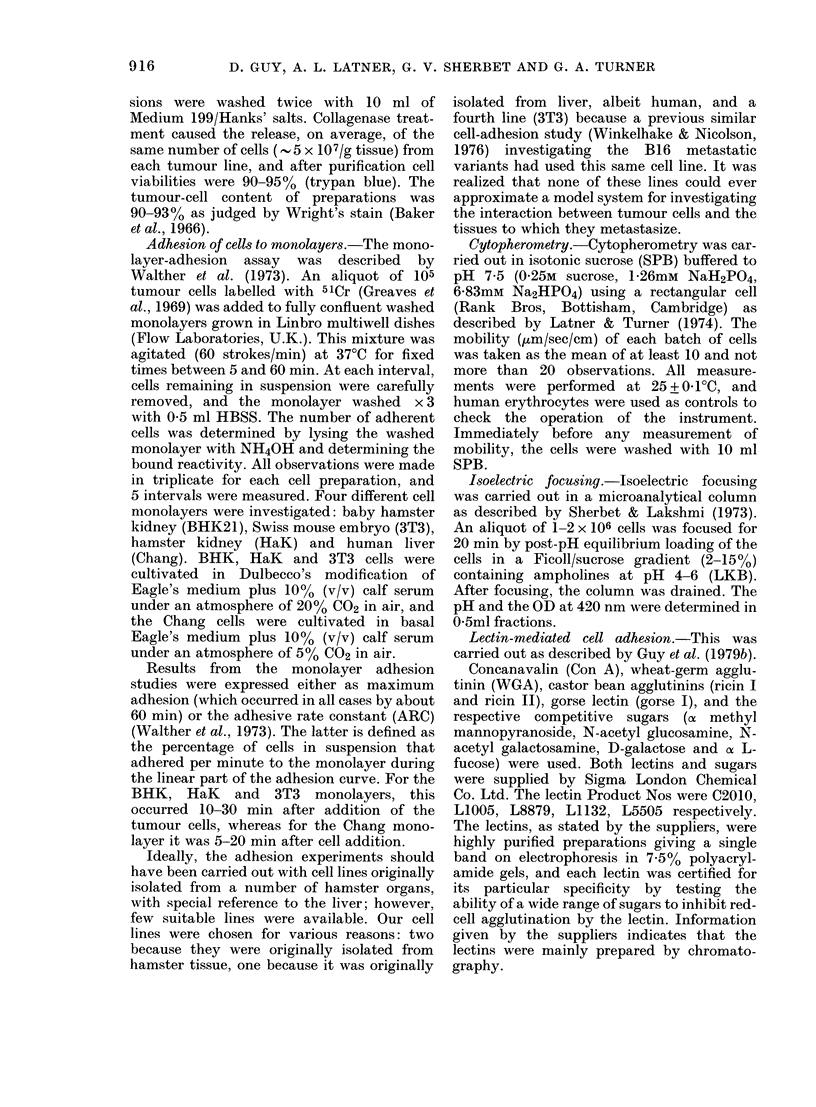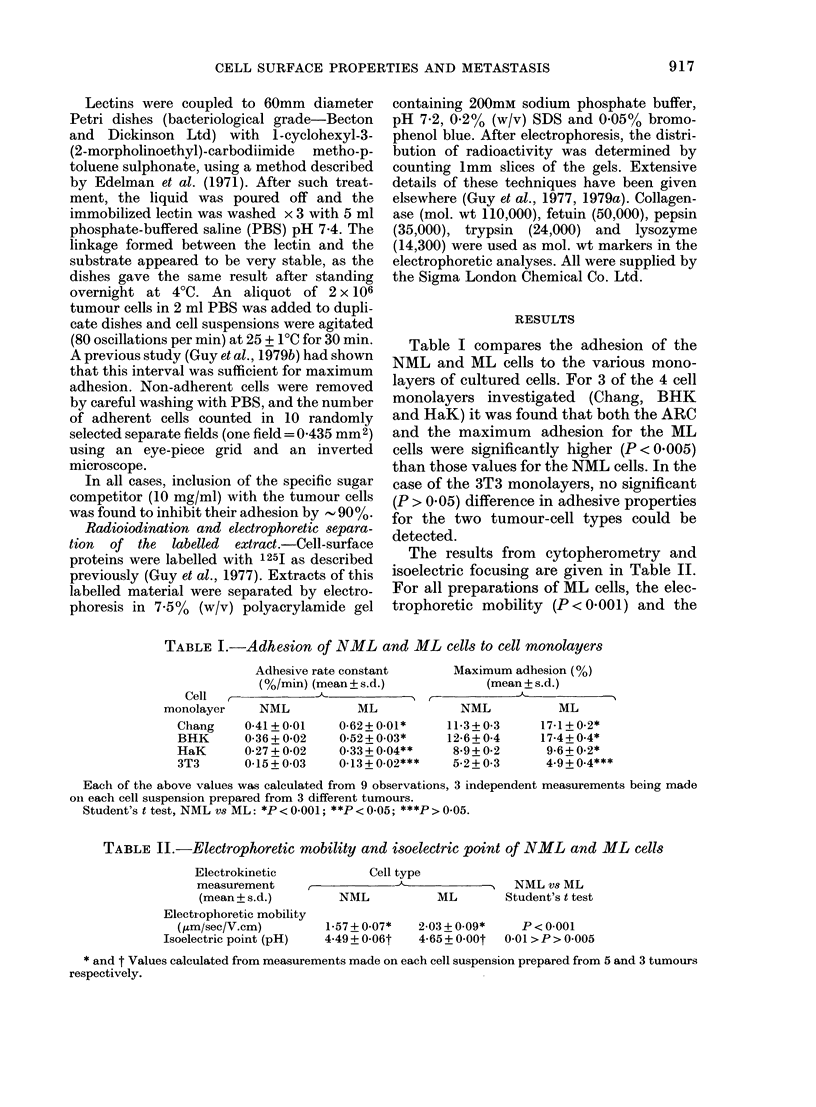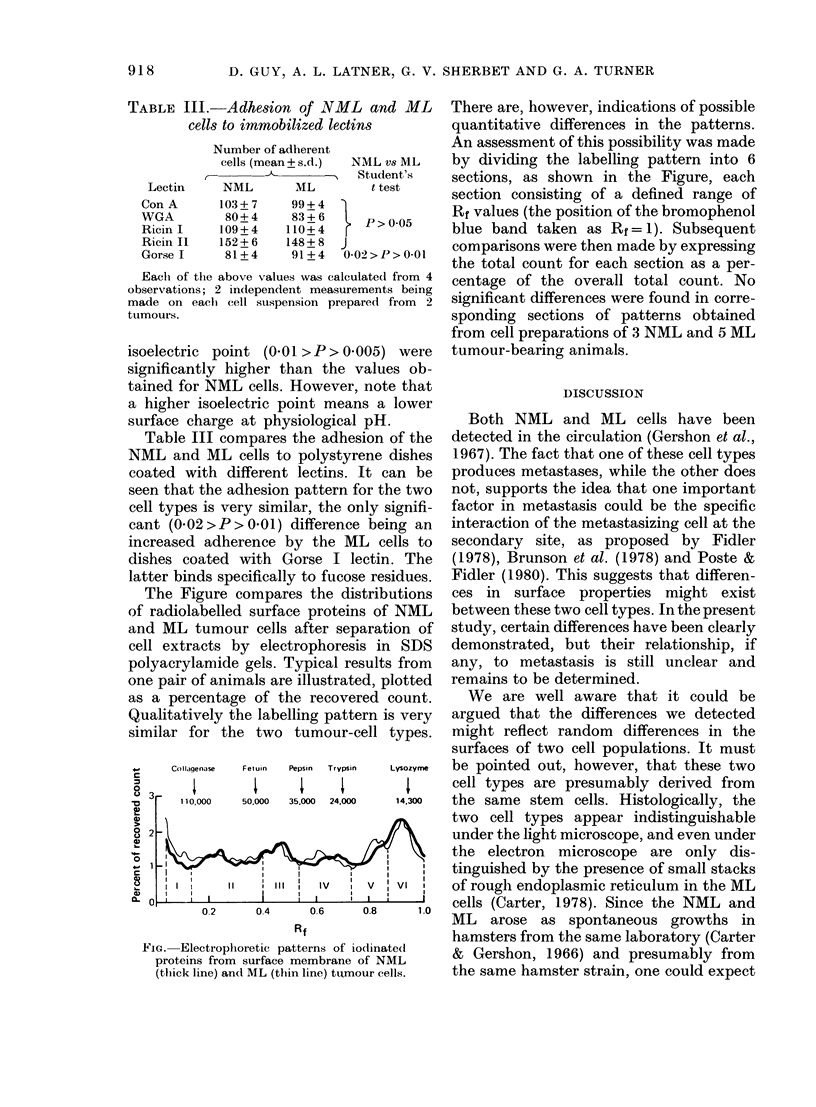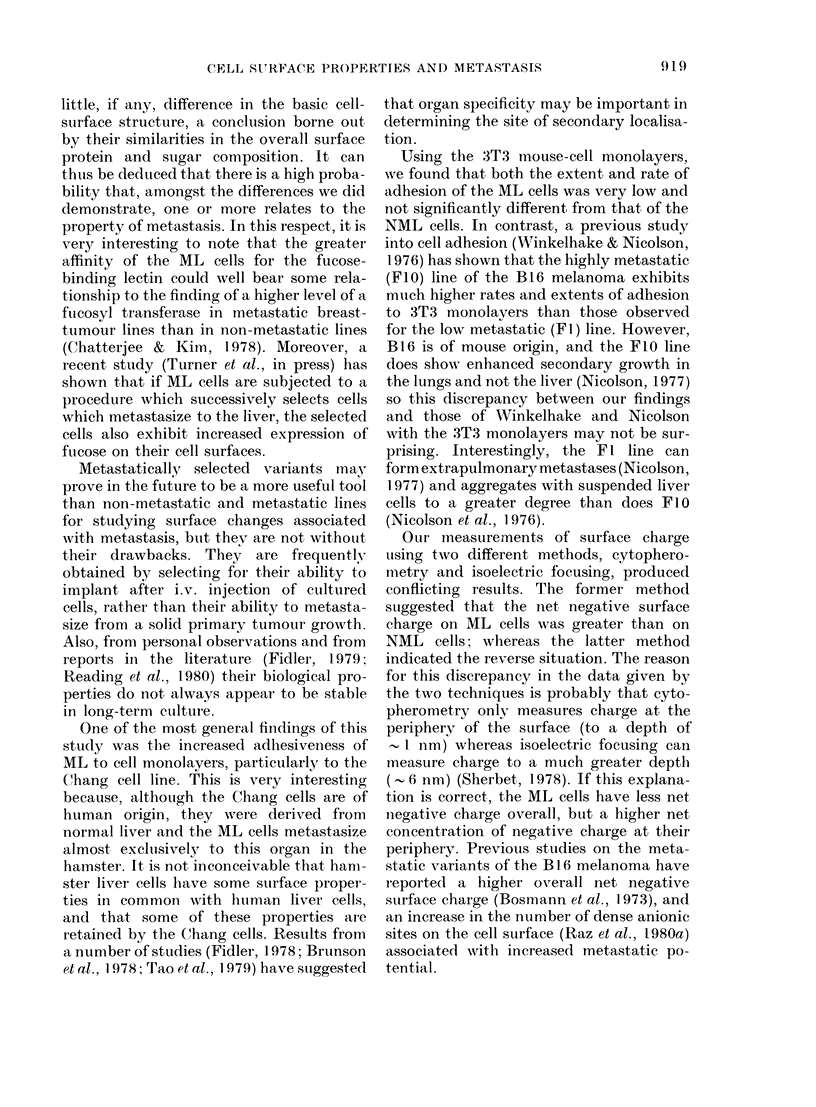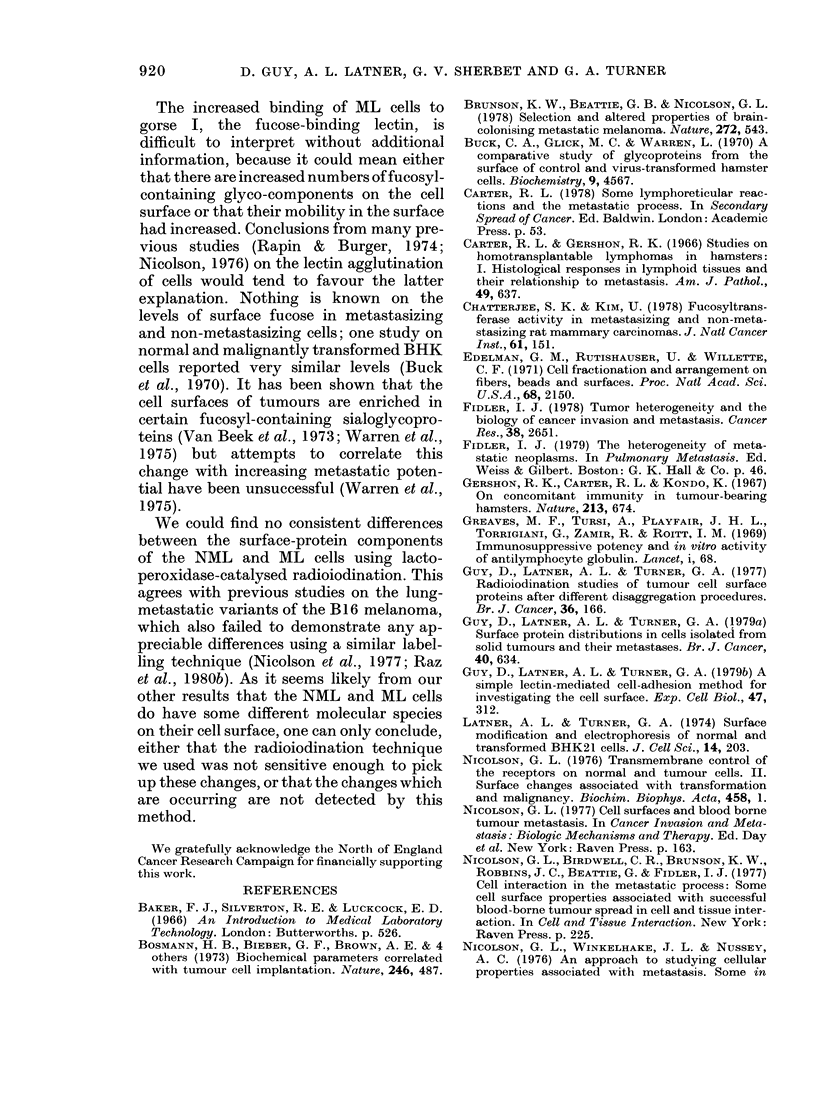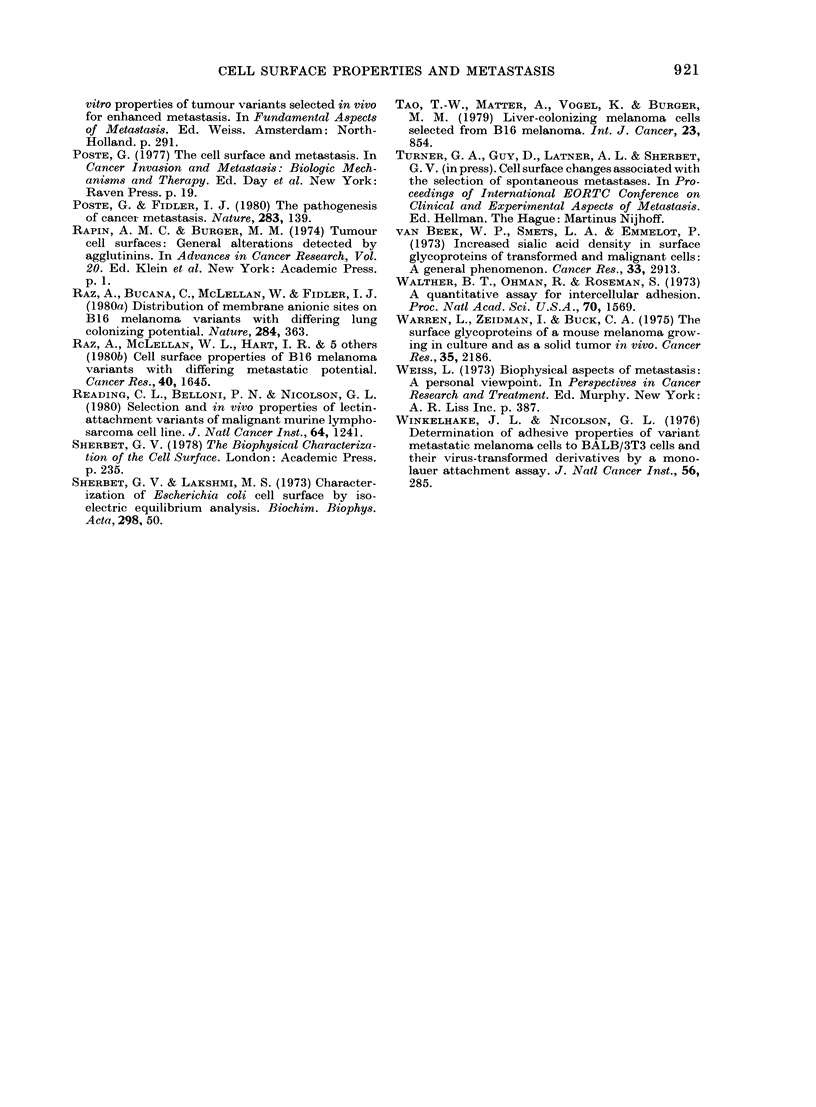# Surface properties of cells isolated from non-metastasizing and metastasizing hamster lymphosarcomas.

**DOI:** 10.1038/bjc.1980.340

**Published:** 1980-12

**Authors:** D. Guy, A. L. Latner, G. V. Sherbet, G. A. Turner

## Abstract

Present evidence suggests that the cell surface has an important role in metastasis. To examine this idea further, the surface properties of single cells isolated from the primary growths of a liver-metastasizing (ML) and a non-metastasizing (NML) lymphosarcoma were compared for adhesion to cell monolayers, cytopherometry, isoelectric focusing, adhesion to immobilized lectins and surface labelling with lactoperoxidase-catalysed radioiodination. It was found that the ML cells had increased adhesion to 3 out of 4 of the monolayers studies; a lower overall surface charge but greater peripheral concentrations of charge; and increased surface expression of the fucose moiety. No consistent difference between the two cell types was detected in the electrophoretic pattern of the labelled surface proteins. These findings are discussed in the light of present knowledge of the cell surface, and it is concluded that the significance of any of the observed changes in relation to metastasis has yet to be established.


*Br. J. Cancer* (1980) **42**, 915

### SURFACE PROPERTIES OF CELLS ISOLATED FROM NON-METASTASIZING AND METASTASIZING HAMSTER LYMPHOSARCOMAS

D. GUY, A. L. LATNER*, G. V. SHERBET AND G. A. TURNER

*From the Cancer Research Unit, University Department of Clinical Biochemistry and Metabolic Medicine, Royal Victoria Infirmary, Newcastle upon Tyne NE1 4LP*

Received 19 March 1980 Accepted 8 September 1980

**Summary.—Present evidence suggests that the cell surface has an important role in metastasis. To examine this idea further, the surface properties of single cells isolated from the primary growths of a liver-metastasizing (ML) and a non-metastasizing (NML) lymphosarcoma were compared for adhesion to cell monolayers, cytopherometry, isoelectric focusing, adhesion to immobilized lectins and surface labelling with lactoperoxidase-catalysed radioiodination. It was found that the ML cells had increased adhesion to 3 out of 4 of the monolayers studied; a lower overall surface charge but greater peripheral concentrations of charge; and increased surface expression of the fucose moiety. No consistent difference between the two cell types was detected in the electrophoretic pattern of the labelled surface proteins. These findings are discussed in the light of present knowledge of the cell surface, and it is concluded that the significance of any of the observed changes in relation to metastasis has yet to be established.**

IT CAN BE INFERRED, on the basis of *in vitro* studies and indirect *in vivo* evidence (Weiss, 1973; Poste, 1977; Nicolson, 1977) that the tumour-cell surface plays an important part in metastasis. If this idea is correct, there should be differences in cell-surface properties between non-metastasizing and metastasizing tumours. The objective of the present study was to examine this possibility. For this purpose, tumour cells were isolated from a non-metastasizing lymphosarcoma and from the primary growth of one which metastasized to the liver: both tumours having the same origin (Carter & Gershon, 1966). The procedures used to prepare these single-cell suspensions had been shown in previous studies to isolate the cells without substantial disruption of their surface structure (Guy *et al.*, 1977; Guy *et al.*, 1979*a*). The methods used to examine the surface properties of the isolated cells were: cell adhesion to cultured monolayers; cytopherometry; isoelectric focusing; cell adhesion to lectin-coated polystyrene; and lactoperoxidase-catalysed radioiodination coupled with gel electrophoresis. It must be emphasized that these methods were adopted to determine differences in surface structure, and did not measure directly any metastatic processes.

### MATERIALS AND METHODS

*Growth of tumours and preparation of cell suspensions.*—Non-metastasizing (NML) and metastasizing (ML) lymphosarcomas were raised in the s.c. site in 2–4-month-old inbred Syrian hamsters. Cell suspensions were prepared using collagenase, and non-viable cells and erythrocytes were removed by centrifugation on Ficoll/Hypaque. These preparative techniques have been previously described in much greater detail (Guy *et al.*, 1977, 1979*a*). All purified single-cell suspen-

* Present address: Microbiological Chemistry Research Laboratory, University of Newcastle upon Tyne.
Correspondence to: Dr G. A. Turner, Cancer Research Unit, University Department of Clinical Biochemistry and Metabolic Medicine, Royal Victoria Infirmary, Newcastle upon Tyne.

sions were washed twice with 10 ml of Medium 199/Hanks' salts. Collagenase treatment caused the release, on average, of the same number of cells ($\sim 5 \times 10^7$/g tissue) from each tumour line, and after purification cell viabilities were 90–95% (trypan blue). The tumour-cell content of preparations was 90–93% as judged by Wright's stain (Baker *et al.*, 1966).

*Adhesion of cells to monolayers.*—The monolayer-adhesion assay was described by Walther *et al.* (1973). An aliquot of $10^5$ tumour cells labelled with $^{51}Cr$ (Greaves *et al.*, 1969) was added to fully confluent washed monolayers grown in Linbro multiwell dishes (Flow Laboratories, U.K.). This mixture was agitated (60 strokes/min) at 37°C for fixed times between 5 and 60 min. At each interval, cells remaining in suspension were carefully removed, and the monolayer washed $\times 3$ with 0·5 ml HBSS. The number of adherent cells was determined by lysing the washed monolayer with $NH_4OH$ and determining the bound reactivity. All observations were made in triplicate for each cell preparation, and 5 intervals were measured. Four different cell monolayers were investigated: baby hamster kidney (BHK21), Swiss mouse embryo (3T3), hamster kidney (HaK) and human liver (Chang). BHK, HaK and 3T3 cells were cultivated in Dulbecco's modification of Eagle's medium plus 10% (v/v) calf serum under an atmosphere of 20% $CO_2$ in air, and the Chang cells were cultivated in basal Eagle's medium plus 10% (v/v) calf serum under an atmosphere of 5% $CO_2$ in air.

Results from the monolayer adhesion studies were expressed either as maximum adhesion (which occurred in all cases by about 60 min) or the adhesive rate constant (ARC) (Walther *et al.*, 1973). The latter is defined as the percentage of cells in suspension that adhered per minute to the monolayer during the linear part of the adhesion curve. For the BHK, HaK and 3T3 monolayers, this occurred 10–30 min after addition of the tumour cells, whereas for the Chang monolayer it was 5–20 min after cell addition.

Ideally, the adhesion experiments should have been carried out with cell lines originally isolated from a number of hamster organs, with special reference to the liver; however, few suitable lines were available. Our cell lines were chosen for various reasons: two because they were originally isolated from hamster tissue, one because it was originally isolated from liver, albeit human, and a fourth line (3T3) because a previous similar cell-adhesion study (Winkelhake & Nicolson, 1976) investigating the B16 metastatic variants had used this same cell line. It was realized that none of these lines could ever approximate a model system for investigating the interaction between tumour cells and the tissues to which they metastasize.

*Cytopherometry.*—Cytopherometry was carried out in isotonic sucrose (SPB) buffered to pH 7·5 (0·25M sucrose, 1·26mM $NaH_2PO_4$, 6·83mM $Na_2HPO_4$) using a rectangular cell (Rank Bros, Bottisham, Cambridge) as described by Latner & Turner (1974). The mobility (μm/sec/cm) of each batch of cells was taken as the mean of at least 10 and not more than 20 observations. All measurements were performed at $25 \pm 0{\cdot}1$°C, and human erythrocytes were used as controls to check the operation of the instrument. Immediately before any measurement of mobility, the cells were washed with 10 ml SPB.

*Isoelectric focusing.*—Isoelectric focusing was carried out in a microanalytical column as described by Sherbet & Lakshmi (1973). An aliquot of $1\text{–}2 \times 10^6$ cells was focused for 20 min by post-pH equilibrium loading of the cells in a Ficoll/sucrose gradient (2–15%) containing ampholines at pH 4–6 (LKB). After focusing, the column was drained. The pH and the OD at 420 nm were determined in 0·5ml fractions.

*Lectin-mediated cell adhesion.*—This was carried out as described by Guy *et al.* (1979*b*).

Concanavalin (Con A), wheat-germ agglutinin (WGA), castor bean agglutinins (ricin I and ricin II), gorse lectin (gorse I), and the respective competitive sugars (α methyl mannopyranoside, N-acetyl glucosamine, N-acetyl galactosamine, D-galactose and α L-fucose) were used. Both lectins and sugars were supplied by Sigma London Chemical Co. Ltd. The lectin Product Nos were C2010, L1005, L8879, L1132, L5505 respectively. The lectins, as stated by the suppliers, were highly purified preparations giving a single band on electrophoresis in 7·5% polyacrylamide gels, and each lectin was certified for its particular specificity by testing the ability of a wide range of sugars to inhibit red-cell agglutination by the lectin. Information given by the suppliers indicates that the lectins were mainly prepared by chromatography.

Lectins were coupled to 60mm diameter Petri dishes (bacteriological grade—Becton and Dickinson Ltd) with 1-cyclohexyl-3-(2-morpholinoethyl)-carbodiimide metho-p-toluene sulphonate, using a method described by Edelman *et al.* (1971). After such treatment, the liquid was poured off and the immobilized lectin was washed ×3 with 5 ml phosphate-buffered saline (PBS) pH 7·4. The linkage formed between the lectin and the substrate appeared to be very stable, as the dishes gave the same result after standing overnight at 4°C. An aliquot of $2 \times 10^6$ tumour cells in 2 ml PBS was added to duplicate dishes and cell suspensions were agitated (80 oscillations per min) at 25 ± 1°C for 30 min. A previous study (Guy *et al.*, 1979*b*) had shown that this interval was sufficient for maximum adhesion. Non-adherent cells were removed by careful washing with PBS, and the number of adherent cells counted in 10 randomly selected separate fields (one field = 0·435 $mm^2$) using an eye-piece grid and an inverted microscope.

In all cases, inclusion of the specific sugar competitor (10 mg/ml) with the tumour cells was found to inhibit their adhesion by ~90%.

*Radioiodination and electrophoretic separation of the labelled extract.*—Cell-surface proteins were labelled with $^{125}I$ as described previously (Guy *et al.*, 1977). Extracts of this labelled material were separated by electrophoresis in 7·5% (w/v) polyacrylamide gel containing 200mM sodium phosphate buffer, pH 7·2, 0·2% (w/v) SDS and 0·05% bromophenol blue. After electrophoresis, the distribution of radioactivity was determined by counting 1mm slices of the gels. Extensive details of these techniques have been given elsewhere (Guy *et al.*, 1977, 1979*a*). Collagenase (mol. wt 110,000), fetuin (50,000), pepsin (35,000), trypsin (24,000) and lysozyme (14,300) were used as mol. wt markers in the electrophoretic analyses. All were supplied by the Sigma London Chemical Co. Ltd.

### RESULTS

Table I compares the adhesion of the NML and ML cells to the various monolayers of cultured cells. For 3 of the 4 cell monolayers investigated (Chang, BHK and HaK) it was found that both the ARC and the maximum adhesion for the ML cells were significantly higher ($P < 0·005$) than those values for the NML cells. In the case of the 3T3 monolayers, no significant ($P > 0·05$) difference in adhesive properties for the two tumour-cell types could be detected.

The results from cytopherometry and isoelectric focusing are given in Table II. For all preparations of ML cells, the electrophoretic mobility ($P < 0·001$) and the

TABLE I.—*Adhesion of NML and ML cells to cell monolayers*

| Cell monolayer | Adhesive rate constant (%/min) (mean ± s.d.) | | Maximum adhesion (%) (mean ± s.d.) | |
|---|---|---|---|---|
| | NML | ML | NML | ML |
| Chang | 0·41 ± 0·01 | 0·62 ± 0·01* | 11·3 ± 0·3 | 17·1 ± 0·2* |
| BHK | 0·36 ± 0·02 | 0·52 ± 0·03* | 12·6 ± 0·4 | 17·4 ± 0·4* |
| HaK | 0·27 ± 0·02 | 0·33 ± 0·04** | 8·9 ± 0·2 | 9·6 ± 0·2* |
| 3T3 | 0·15 ± 0·03 | 0·13 ± 0·02*** | 5·2 ± 0·3 | 4·9 ± 0·4*** |

Each of the above values was calculated from 9 observations, 3 independent measurements being made on each cell suspension prepared from 3 different tumours.
Student's *t* test, NML *vs* ML: *$P < 0·001$; **$P < 0·05$; ***$P > 0·05$.

TABLE II.—*Electrophoretic mobility and isoelectric point of NML and ML cells*

| Electrokinetic measurement (mean ± s.d.) | Cell type NML | ML | NML *vs* ML Student's *t* test |
|---|---|---|---|
| Electrophoretic mobility (μm/sec/V.cm) | 1·57 ± 0·07* | 2·03 ± 0·09* | $P < 0·001$ |
| Isoelectric point (pH) | 4·49 ± 0·06† | 4·65 ± 0·00† | $0·01 > P > 0·005$ |

* and † Values calculated from measurements made on each cell suspension prepared from 5 and 3 tumours respectively.

TABLE III.—*Adhesion of NML and ML cells to immobilized lectins*

| Lectin | Number of adherent cells (mean ± s.d.) NML | ML | NML *vs* ML Student's *t* test |
|---|---|---|---|
| Con A | 103 ± 7 | 99 ± 4 | $P > 0.05$ |
| WGA | 80 ± 4 | 83 ± 6 | |
| Ricin I | 109 ± 4 | 110 ± 4 | |
| Ricin II | 152 ± 6 | 148 ± 8 | |
| Gorse I | 81 ± 4 | 91 ± 4 | $0.02 > P > 0.01$ |

Each of the above values was calculated from 4 observations; 2 independent measurements being made on each cell suspension prepared from 2 tumours.

isoelectric point ($0.01 > P > 0.005$) were significantly higher than the values obtained for NML cells. However, note that a higher isoelectric point means a lower surface charge at physiological pH.

Table III compares the adhesion of the NML and ML cells to polystyrene dishes coated with different lectins. It can be seen that the adhesion pattern for the two cell types is very similar, the only significant ($0.02 > P > 0.01$) difference being an increased adherence by the ML cells to dishes coated with Gorse I lectin. The latter binds specifically to fucose residues.

The Figure compares the distributions of radiolabelled surface proteins of NML and ML tumour cells after separation of cell extracts by electrophoresis in SDS polyacrylamide gels. Typical results from one pair of animals are illustrated, plotted as a percentage of the recovered count. Qualitatively the labelling pattern is very similar for the two tumour-cell types.

FIG.—Electrophoretic patterns of iodinated proteins from surface membrane of NML (thick line) and ML (thin line) tumour cells.

There are, however, indications of possible quantitative differences in the patterns. An assessment of this possibility was made by dividing the labelling pattern into 6 sections, as shown in the Figure, each section consisting of a defined range of $R_f$ values (the position of the bromophenol blue band taken as $R_f = 1$). Subsequent comparisons were then made by expressing the total count for each section as a percentage of the overall total count. No significant differences were found in corresponding sections of patterns obtained from cell preparations of 3 NML and 5 ML tumour-bearing animals.

### DISCUSSION

Both NML and ML cells have been detected in the circulation (Gershon *et al.*, 1967). The fact that one of these cell types produces metastases, while the other does not, supports the idea that one important factor in metastasis could be the specific interaction of the metastasizing cell at the secondary site, as proposed by Fidler (1978), Brunson *et al.* (1978) and Poste & Fidler (1980). This suggests that differences in surface properties might exist between these two cell types. In the present study, certain differences have been clearly demonstrated, but their relationship, if any, to metastasis is still unclear and remains to be determined.

We are well aware that it could be argued that the differences we detected might reflect random differences in the surfaces of two cell populations. It must be pointed out, however, that these two cell types are presumably derived from the same stem cells. Histologically, the two cell types appear indistinguishable under the light microscope, and even under the electron microscope are only distinguished by the presence of small stacks of rough endoplasmic reticulum in the ML cells (Carter, 1978). Since the NML and ML arose as spontaneous growths in hamsters from the same laboratory (Carter & Gershon, 1966) and presumably from the same hamster strain, one could expect little, if any, difference in the basic cell-surface structure, a conclusion borne out by their similarities in the overall surface protein and sugar composition. It can thus be deduced that there is a high probability that, amongst the differences we did demonstrate, one or more relates to the property of metastasis. In this respect, it is very interesting to note that the greater affinity of the ML cells for the fucose-binding lectin could well bear some relationship to the finding of a higher level of a fucosyl transferase in metastatic breast-tumour lines than in non-metastatic lines (Chatterjee & Kim, 1978). Moreover, a recent study (Turner *et al.*, in press) has shown that if ML cells are subjected to a procedure which successively selects cells which metastasize to the liver, the selected cells also exhibit increased expression of fucose on their cell surfaces.

Metastatically selected variants may prove in the future to be a more useful tool than non-metastatic and metastatic lines for studying surface changes associated with metastasis, but they are not without their drawbacks. They are frequently obtained by selecting for their ability to implant after i.v. injection of cultured cells, rather than their ability to metastasize from a solid primary tumour growth. Also, from personal observations and from reports in the literature (Fidler, 1979; Reading *et al.*, 1980) their biological properties do not always appear to be stable in long-term culture.

One of the most general findings of this study was the increased adhesiveness of ML to cell monolayers, particularly to the Chang cell line. This is very interesting because, although the Chang cells are of human origin, they were derived from normal liver and the ML cells metastasize almost exclusively to this organ in the hamster. It is not inconceivable that hamster liver cells have some surface properties in common with human liver cells, and that some of these properties are retained by the Chang cells. Results from a number of studies (Fidler, 1978; Brunson *et al.*, 1978; Tao *et al.*, 1979) have suggested that organ specificity may be important in determining the site of secondary localisation.

Using the 3T3 mouse-cell monolayers, we found that both the extent and rate of adhesion of the ML cells was very low and not significantly different from that of the NML cells. In contrast, a previous study into cell adhesion (Winkelhake & Nicolson, 1976) has shown that the highly metastatic (F10) line of the B16 melanoma exhibits much higher rates and extents of adhesion to 3T3 monolayers than those observed for the low metastatic (F1) line. However, B16 is of mouse origin, and the F10 line does show enhanced secondary growth in the lungs and not the liver (Nicolson, 1977) so this discrepancy between our findings and those of Winkelhake and Nicolson with the 3T3 monolayers may not be surprising. Interestingly, the F1 line can form extrapulmonary metastases (Nicolson, 1977) and aggregates with suspended liver cells to a greater degree than does F10 (Nicolson *et al.*, 1976).

Our measurements of surface charge using two different methods, cytopherometry and isoelectric focusing, produced conflicting results. The former method suggested that the net negative surface charge on ML cells was greater than on NML cells; whereas the latter method indicated the reverse situation. The reason for this discrepancy in the data given by the two techniques is probably that cytopherometry only measures charge at the periphery of the surface (to a depth of $\sim 1$ nm) whereas isoelectric focusing can measure charge to a much greater depth ($\sim 6$ nm) (Sherbet, 1978). If this explanation is correct, the ML cells have less net negative charge overall, but a higher net concentration of negative charge at their periphery. Previous studies on the metastatic variants of the B16 melanoma have reported a higher overall net negative surface charge (Bosmann *et al.*, 1973), and an increase in the number of dense anionic sites on the cell surface (Raz *et al.*, 1980*a*) associated with increased metastatic potential.

The increased binding of ML cells to gorse I, the fucose-binding lectin, is difficult to interpret without additional information, because it could mean either that there are increased numbers of fucosyl-containing glyco-components on the cell surface or that their mobility in the surface had increased. Conclusions from many previous studies (Rapin & Burger, 1974; Nicolson, 1976) on the lectin agglutination of cells would tend to favour the latter explanation. Nothing is known on the levels of surface fucose in metastasizing and non-metastasizing cells; one study on normal and malignantly transformed BHK cells reported very similar levels (Buck *et al.*, 1970). It has been shown that the cell surfaces of tumours are enriched in certain fucosyl-containing sialoglycoproteins (Van Beek *et al.*, 1973; Warren *et al.*, 1975) but attempts to correlate this change with increasing metastatic potential have been unsuccessful (Warren *et al.*, 1975).

We could find no consistent differences between the surface-protein components of the NML and ML cells using lactoperoxidase-catalysed radioiodination. This agrees with previous studies on the lung-metastatic variants of the B16 melanoma, which also failed to demonstrate any appreciable differences using a similar labelling technique (Nicolson *et al.*, 1977; Raz *et al.*, 1980*b*). As it seems likely from our other results that the NML and ML cells do have some different molecular species on their cell surface, one can only conclude, either that the radioiodination technique we used was not sensitive enough to pick up these changes, or that the changes which are occurring are not detected by this method.

We gratefully acknowledge the North of England Cancer Research Campaign for financially supporting this work.

### REFERENCES

BAKER, F. J., SILVERTON, R. E. & LUCKCOCK, E. D. (1966) *An Introduction to Medical Laboratory Technology*. London: Butterworths. p. 526.

BOSMANN, H. B., BIEBER, G. F., BROWN, A. E. & 4 others (1973) Biochemical parameters correlated with tumour cell implantation. *Nature*, **246**, 487.

BRUNSON, K. W., BEATTIE, G. B. & NICOLSON, G. L. (1978) Selection and altered properties of brain-colonising metastatic melanoma. *Nature*, **272**, 543.

BUCK, C. A., GLICK, M. C. & WARREN, L. (1970) A comparative study of glycoproteins from the surface of control and virus-transformed hamster cells. *Biochemistry*, **9**, 4567.

CARTER, R. L. (1978) Some lymphoreticular reactions and the metastatic process. In *Secondary Spread of Cancer*. Ed. Baldwin. London: Academic Press. p. 53.

CARTER, R. L. & GERSHON, R. K. (1966) Studies on homotransplantable lymphomas in hamsters: I. Histological responses in lymphoid tissues and their relationship to metastasis. *Am. J. Pathol.*, **49**, 637.

CHATTERJEE, S. K. & KIM, U. (1978) Fucosyltransferase activity in metastasizing and non-metastasizing rat mammary carcinomas. *J. Natl Cancer Inst.*, **61**, 151.

EDELMAN, G. M., RUTISHAUSER, U. & WILLETTE, C. F. (1971) Cell fractionation and arrangement on fibers, beads and surfaces. *Proc. Natl Acad. Sci. U.S.A.*, **68**, 2150.

FIDLER, I. J. (1978) Tumor heterogeneity and the biology of cancer invasion and metastasis. *Cancer Res.*, **38**, 2651.

FIDLER, I. J. (1979) The heterogeneity of metastatic neoplasms. In *Pulmonary Metastasis*. Ed. Weiss & Gilbert. Boston: G. K. Hall & Co. p. 46.

GERSHON, R. K., CARTER, R. L. & KONDO, K. (1967) On concomitant immunity in tumour-bearing hamsters. *Nature*, **213**, 674.

GREAVES, M. F., TURSI, A., PLAYFAIR, J. H. L., TORRIGIANI, G., ZAMIR, R. & ROITT, I. M. (1969) Immunosuppressive potency and *in vitro* activity of antilymphocyte globulin. *Lancet*, i, 68.

GUY, D., LATNER, A. L. & TURNER, G. A. (1977) Radioiodination studies of tumour cell surface proteins after different disaggregation procedures. *Br. J. Cancer*, **36**, 166.

GUY, D., LATNER, A. L. & TURNER, G. A. (1979*a*) Surface protein distributions in cells isolated from solid tumours and their metastases. *Br. J. Cancer*, **40**, 634.

GUY, D., LATNER, A. L. & TURNER, G. A. (1979*b*) A simple lectin-mediated cell-adhesion method for investigating the cell surface. *Exp. Cell Biol.*, **47**, 312.

LATNER, A. L. & TURNER, G. A. (1974) Surface modification and electrophoresis of normal and transformed BHK21 cells. *J. Cell Sci.*, **14**, 203.

NICOLSON, G. L. (1976) Transmembrane control of the receptors on normal and tumour cells. II. Surface changes associated with transformation and malignancy. *Biochim. Biophys. Acta*, **458**, 1.

NICOLSON, G. L. (1977) Cell surfaces and blood borne tumour metastasis. In *Cancer Invasion and Metastasis: Biologic Mechanisms and Therapy*. Ed. Day *et al.* New York: Raven Press. p. 163.

NICOLSON, G. L., BIRDWELL, C. R., BRUNSON, K. W., ROBBINS, J. C., BEATTIE, G. & FIDLER, I. J. (1977) Cell interaction in the metastatic process: Some cell surface properties associated with successful blood-borne tumour spread in cell and tissue interaction. In *Cell and Tissue Interaction*. New York: Raven Press. p. 225.

NICOLSON, G. L., WINKELHAKE, J. L. & NUSSEY, A. C. (1976) An approach to studying cellular properties associated with metastasis. Some *in*

*vitro* properties of tumour variants selected *in vivo* for enhanced metastasis. In *Fundamental Aspects of Metastasis*. Ed. Weiss. Amsterdam: North-Holland. p. 291.

POSTE, G. (1977) The cell surface and metastasis. In *Cancer Invasion and Metastasis: Biologic Mechanisms and Therapy*. Ed. Day *et al.* New York: Raven Press. p. 19.

POSTE, G. & FIDLER, I. J. (1980) The pathogenesis of cancer metastasis. *Nature*, **283**, 139.

RAPIN, A. M. C. & BURGER, M. M. (1974) Tumour cell surfaces: General alterations detected by agglutinins. In *Advances in Cancer Research, Vol. 20*. Ed. Klein *et al.* New York: Academic Press. p. 1.

RAZ, A., BUCANA, C., MCLELLAN, W. & FIDLER, I. J. (1980*a*) Distribution of membrane anionic sites on B16 melanoma variants with differing lung colonizing potential. *Nature*, **284**, 363.

RAZ, A., MCLELLAN, W. L., HART, I. R. & 5 others (1980*b*) Cell surface properties of B16 melanoma variants with differing metastatic potential. *Cancer Res.*, **40**, 1645.

READING, C. L., BELLONI, P. N. & NICOLSON, G. L. (1980) Selection and *in vivo* properties of lectin-attachment variants of malignant murine lymphosarcoma cell line. *J. Natl Cancer Inst.*, **64**, 1241.

SHERBET, G. V. (1978) *The Biophysical Characterization of the Cell Surface*. London: Academic Press. p. 235.

SHERBET, G. V. & LAKSHMI, M. S. (1973) Characterization of *Escherichia coli* cell surface by isoelectric equilibrium analysis. *Biochim. Biophys. Acta*, **298**, 50.

TAO, T.-W., MATTER, A., VOGEL, K. & BURGER, M. M. (1979) Liver-colonizing melanoma cells selected from B16 melanoma. *Int. J. Cancer*, **23**, 854.

TURNER, G. A., GUY, D., LATNER, A. L. & SHERBET, G. V. (in press). Cell surface changes associated with the selection of spontaneous metastases. In *Proceedings of International EORTC Conference on Clinical and Experimental Aspects of Metastasis*. Ed. Hellman. The Hague: Martinus Nijhoff.

VAN BEEK, W. P., SMETS, L. A. & EMMELOT, P. (1973) Increased sialic acid density in surface glycoproteins of transformed and malignant cells: A general phenomenon. *Cancer Res.*, **33**, 2913.

WALTHER, B. T., OHMAN, R. & ROSEMAN, S. (1973) A quantitative assay for intercellular adhesion. *Proc. Natl Acad. Sci. U.S.A.*, **70**, 1569.

WARREN, L., ZEIDMAN, I. & BUCK, C. A. (1975) The surface glycoproteins of a mouse melanoma growing in culture and as a solid tumor *in vivo*. *Cancer Res.*, **35**, 2186.

WEISS, L. (1973) Biophysical aspects of metastasis: A personal viewpoint. In *Perspectives in Cancer Research and Treatment*. Ed. Murphy. New York: A. R. Liss Inc. p. 387.

WINKELHAKE, J. L. & NICOLSON, G. L. (1976) Determination of adhesive properties of variant metastatic melanoma cells to BALB/3T3 cells and their virus-transformed derivatives by a monolauer attachment assay. *J. Natl Cancer Inst.*, **56**, 285.